# Distal Lung Microenvironment Triggers Release of Mediators Recognized as Potential Systemic Biomarkers for Idiopathic Pulmonary Fibrosis

**DOI:** 10.3390/ijms222413421

**Published:** 2021-12-14

**Authors:** Dimitrios Kalafatis, Anna Löfdahl, Per Näsman, Göran Dellgren, Åsa M. Wheelock, Linda Elowsson Rendin, Magnus Sköld, Gunilla Westergren-Thorsson

**Affiliations:** 1 Respiratory Medicine Unit, Department of Medicine Solna and Center for Molecular Medicine, Karolinska Institutet, SE-171 76 Stockholm, Sweden; asa.wheelock@ki.se (Å.M.W.); magnus.skold@ki.se (M.S.); 2 Department of Experimental Medical Science, Lung Biology, Lund University, SE-221 84 Lund, Sweden; anna.lofdahl@med.lu.se (A.L.); linda.elowsson_rendin@med.lu.se (L.E.R.); gunilla.westergren-thorsson@med.lu.se (G.W.-T.); 3 Center for Safety Research, KTH, Royal Institute of Technology, SE-100 44 Stockholm, Sweden; per.nasman@abe.kth.se; 4 Department of Cardiothoracic Surgery and Transplant Institute, Sahlgrenska University Hospital, SE-413 45 Gothenburg, Sweden; goran.dellgren@vgregion.se; 5 Department of Respiratory Medicine and Allergy, Karolinska University Hospital, SE-171 76 Stockholm, Sweden

**Keywords:** idiopathic pulmonary fibrosis, biomarkers, fibroblast, extracellular matrix

## Abstract

Idiopathic pulmonary fibrosis (IPF) is a progressive fibrotic lung disease with an unmet need of biomarkers that can aid in the diagnostic and prognostic assessment of the disease and response to treatment. In this two-part explorative proteomic study, we demonstrate how proteins associated with tissue remodeling, inflammation and chemotaxis such as MMP7, CXCL13 and CCL19 are released in response to aberrant extracellular matrix (ECM) in IPF lung. We used a novel ex vivo model where decellularized lung tissue from IPF patients and healthy donors were repopulated with healthy fibroblasts to monitor locally released mediators. Results were validated in longitudinally collected serum samples from 38 IPF patients and from 77 healthy controls. We demonstrate how proteins elevated in the ex vivo model (e.g., MMP7), and other serum proteins found elevated in IPF patients such as HGF, VEGFA, MCP-3, IL-6 and TNFRSF12A, are associated with disease severity and progression and their response to antifibrotic treatment. Our study supports the model’s applicability in studying mechanisms involved in IPF and provides additional evidence for both established and potentially new biomarkers in IPF.

## 1. Introduction

Idiopathic pulmonary fibrosis (IPF) is an irreversible interstitial pneumonia of progressive nature. Patients with IPF usually present exertional dyspnea and cough that over time ultimately result in restricted ventilation, hypoxemia, respiratory failure and death within a few years from diagnosis [1]. Diagnosing and managing IPF requires extensive resources and a multidisciplinary approach including radiological, physiological and histopathological examinations. The variability in disease course is evident in IPF where some patients demonstrate a slow decline in lung function, while others present an accelerated disease progression or even fatal respiratory insufficiency [2]. The heterogeneity of IPF creates difficulties in predicting disease course, warranting the need for effective diagnostic and prognostic biomarkers. So far, there are no treatments available that can reverse or halt the progression of fibrosis despite promising results of early drug candidates in preclinical studies. Both in vitro and in vivo models have not fully recapitulated the complex fibrogenic activity that occurs in the lungs of IPF patients. Thus, there is a need for preclinical models mimicking events in the fibrotic processes that may help in identifying key disease mechanisms and possible therapeutic targets. 

Several growth factors, cytokines and chemokines involved in regulating coagulation, angiogenesis, inflammation and repair responses have shown to be implicated in the pathogenesis of IPF in a number of preclinical and clinical studies [3,4,5,6,7,8,9,10]. Their impact on biological pathways steers cellular responses in IPF where activation of alveolar epithelial cells, formation of fibroblast foci and aberrant extracellular matrix (ECM) deposition result in remodeling of lung parenchyma [11]. However, the underlying disease mechanism driving the continuous cellular activation and the increased deposition of ECM proteins in IPF is still not fully understood. Fibroblasts are one of the main ECM-producing cells and have been recognized as one of the most important targets in fibrotic development, contributing to the detrimental remodeling of IPF lung tissue [12]. The lung tissue in IPF is densely packed with connective tissue with elevated levels of collagens, proteoglycans and glycoproteins [13]. The thickened alveolar interstitial space with distorted epithelium and vascular architecture creates a unique ECM-niche that impacts resident cells of the lung [14,15]. Additionally, the biomechanical properties of the fibrotic tissue exert a major impact on cellular behavior including cell proliferation, migration and differentiation [13,16,17]. The stiffness of the fibrotic tissue actively contributes to disease progression in IPF altering gene expression profiles and translational processes in fibroblasts and the induction of mechanotransduction pathways [18,19,20,21]. As fibroblasts are constantly sensing their microenvironment, they are highly responsive to pathological changes, and the surrounding cell microenvironment has been shown to be more influential on cellular behavior than cellular origin [13,21], emphasizing the importance of cell-ECM crosstalk.

Decellularized lung tissue with retained material properties offers an opportunity to study interactions between cells and ECM of the lung in health and disease. We have created a novel ex vivo model that mimics the structural and biomechanical properties of the lung-ECM by using decellularized tissue, devoid of cellular content, from healthy donors and IPF patients [13]. By repopulating the lung-ECM (aka scaffolds) with healthy human fibroblasts we have created a preclinical model to study how cells respond in an IPF lung. By monitoring released cell mediators over time, we have discovered an IPF-associated protein profile that demonstrates an upregulation of growth factors, cytokines and chemokines involved in inflammation and tissue remodeling. 

This translational study serves two purposes—to study potential biomarkers associated with tissue remodeling, inflammation and chemotaxis by using an ex vivo model, and to analyze these candidate biomarkers in serum from IPF patients and investigate their possible associations to disease severity and progression. We hypothesize that the microenvironment of the lung in IPF governs cellular activity including release of mediators that is reflected in the blood circulation and is associated to disease activity and progression. 

## 2. Results

### 2.1. Proteins Released in Ex Vivo Model

The repopulated scaffolds derived from end-stage IPF patients and healthy individuals exhibited preserved tissue morphology and viable cell cultures as previously described by Elowsson Rendin et al. [13]. The fibrotic lung-ECM triggered an altered cellular activation over time as seen in measured protein levels in collected cell culture medium. We detected 12 proteins elevated in repopulated IPF scaffolds (Table 1), two of which, MMP7 and CXCL13, demonstrated the largest increase compared to repopulated healthy scaffolds. 

**Figure 1:**
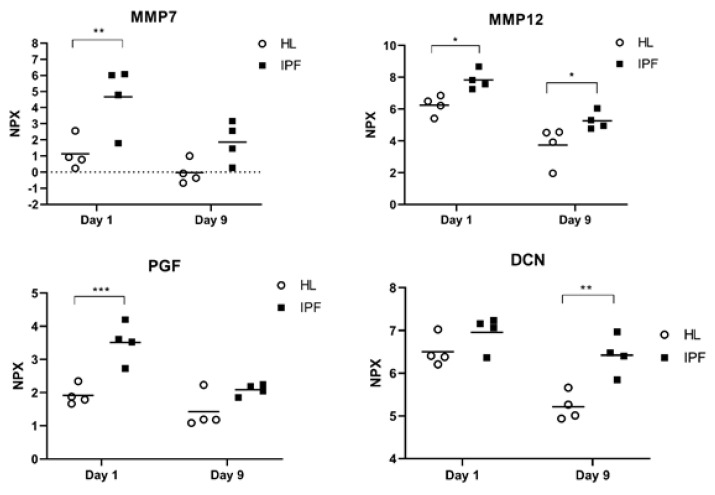
Remodeling proteins in IPF repopulated scaffolds. Relative protein amount (NPX) of proteins associated to remodeling of ECM, measured in cell medium from repopulated scaffolds derived from idiopathic pulmonary fibrosis (IPF) patients or healthy (HL) individuals following 1 day and 9 days in culture. Dotted line = limit of detection, y = 0. Two-way ANOVA with Sidak’s multiple comparison test. * *p* < 0.05, ** *p* < 0.01, *** *p* < 0.005.

Four (MMP7, MMP12, DCN and PGF) (Figure 1) of the 12 elevated proteins are known to regulate tissue remodeling while the other eight are associated in processes with inflammation and chemotaxis. MMP12, GAL9 and CXCL13 showed significantly elevated levels in IPF at both day 1 and day 9 in culture. The number of released proteins at day 9 were maintained or reduced in healthy and in IPF scaffolds in comparison to day 1, where the IPF scaffolds generated a significant temporal reduction in all proteins, except for DCN.

### 2.2. Characteristics of IPF Patients and Controls 

Analysis of demographic and clinical data of the enrolled patients showed that IPF patients were older, a majority men with a history of smoking, with a restrictive lung function and low DLCO (Table 2). 

Classification according to Gender-Age-Physiology stage (GAP), a staging system providing the average risk of mortality in IPF-patients [22], and CPI revealed that more than half of the patients (55%) were classified as GAP-stage 1 and the remaining as GAP-stage 2 (45%), while their CPI was 43.0 + 10.7. Twelve (32%) patients were on antifibrotic treatment at both baseline and follow up sampling. Meanwhile, 13 (34%) patients were put on treatment after the baseline sampling and remained treated at follow up. Eleven (29%) patients remained untreated throughout the whole study, while two (5%) patients terminated treatment after baseline. Outcome of death or lung transplantation in IPF patients were followed from baseline until December 2020 (median time: 43 months (min-max: 25–52 months)), and occurred in eight patients, with the second serum sample taken at a median time of 20 months (range 10–32 months) prior to outcome. Subjects in the control cohort had equal proportions of both males and females and current and never smokers (Table 2).

### 2.3. Proteins in Baseline Serum from IPF Patients Compared to Controls 

**Figure 2:**
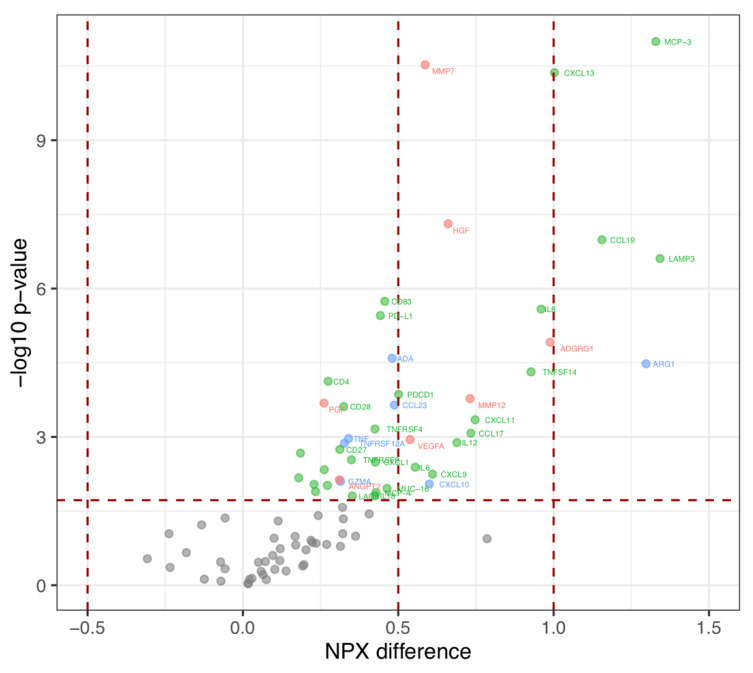
Levels of circulating proteins in IPF patients versus controls. Volcano plot of the difference in relative protein amount (NPX) (x-axis) by log10 of *p*-value. Red: proteins associated with tissue remodeling; Green: proteins associated with inflammation/chemotaxis; Blue: proteins with overlapping functions. One-way ANOVA adjusted for age with Benjamini–Hochberg corrected *p*-value to control a false discovery rate at 5%.

We identified 44 proteins with elevated levels in the baseline samples (corrected *p*-value < 0.05, ANOVA adjusted for age) (Figure 2). 

Many of the proteins (30 out of 44) regulated inflammation and chemotaxis. Five of these (MCP-3, CXCL13, CCL19, LAMP3 and ARG1) had a NPX-difference > 1, i.e., a doubling of protein expression between groups, and 14 proteins had a 1 < NPX-difference > 0.5, meaning an increased protein level of more than 50% (Table 3). Among the proteins with highest expression in IPF compared to healthy controls were also ADGRG1, IL8 and TNFSF14 with an NPX-difference > 0.9. Ten of the elevated proteins identified in the ex vivo model were also elevated in IPF at baseline (Table 3) of which three proteins (MMP7, MMP12 and PGF) regulate tissue remodeling processes. Additional remodeling proteins found elevated in IPF serum included VEGFA and HGF. There was no protein with lower level in IPF patients compared to healthy controls. 

**Figure 3:**
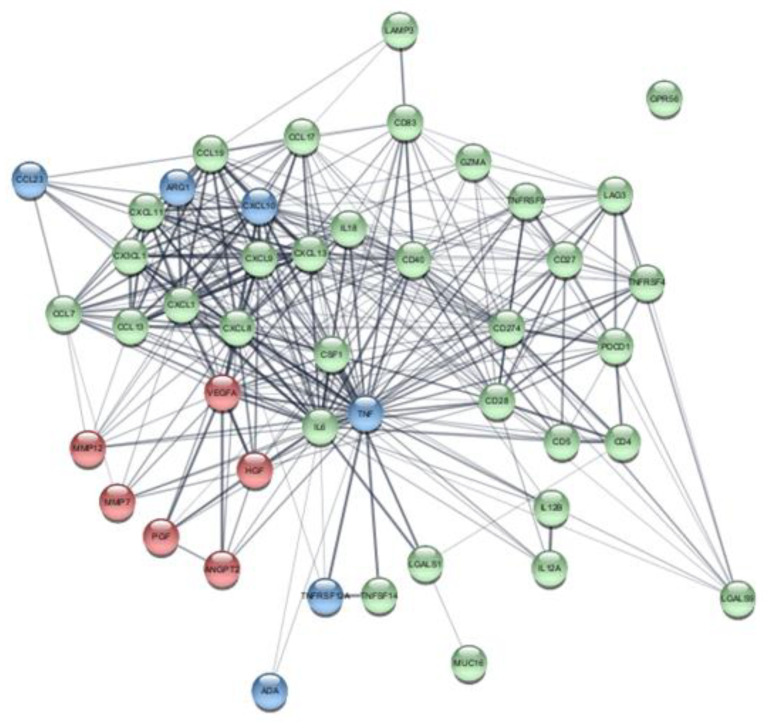
Bioinformatic analysis of elevated proteins in IPF serum. Network of protein-protein interaction of significantly elevated proteins in IPF serum at baseline compared to healthy controls Annotated biological functions are highlighted with color-coded nodes. Nodes categorized according to biological function; Red nodes = tissue remodeling, Green nodes = inflammation/chemotaxis, Blue nodes = overlapping functions.

Bioinformatic analysis of the elevated proteins observed in IPF patients revealed a network of associated protein-protein interactions (Figure 3), with nodes important for tissue remodeling, inflammation/chemotaxis as well as overlapping functions. A cluster of CXC- and CCL chemokine clusters was visualized that connected with IL6, TNF, VEGFA and HGF, which also showed protein interactions with tissue remodeling proteins MMP12, MMP7, PGF and ANGPT2. 

Pathway analysis of all elevated proteins in IPF revealed several significant associations with signaling pathways such as chemokine/cytokine signaling, NF-kB signaling and TNF receptor binding (Figure S1).

A complete list of all proteins and their age-unadjusted protein expressions are available in Table S1. When calculations were done without adjusting for age, 60 proteins were elevated in IPF compared to controls and four proteins (VEGFR-2, ANGPT1, FASLG, PDGF-B) demonstrated decreased levels. Moreover, in this data set all 12 proteins elevated in the ex vivo model in IPF were also significantly increased in serum from IPF patients compared to controls. 

### 2.4. Proteins in IPF Serum at Follow Up 

Thirty proteins demonstrated a significantly altered protein expression in IPF follow up samples compared to healthy controls, out of which 28 proteins were elevated in IPF. Eighteen (Figure S2 and Table S2) were associated to inflammation/chemotaxis, nine with tissue remodeling and three with overlapping functions. Of the 44 proteins that were upregulated at baseline, 24 proteins were also elevated in the follow up samples compared to controls. Levels of 21 proteins were decreased at follow up compared to baseline (Table 4). Proteins with the largest decrement included EGF, FGF2 (regulating tissue remodeling) and CASP-8 (overlapping functions). Only one, PTN, a remodeling protein, showed an increase in protein expression from baseline.

A complete list of all proteins analyzed and their protein amount at follow up is available in Table S3.

### 2.5. Protein Expression and Disease Severity 

To investigate the proteins’ potential in reflecting the severity of IPF, proteins were correlated with common clinical measures of disease severity at both baseline and follow up (Table 5 and Table S4). Several proteins found differentially expressed in IPF compared to controls, were correlated to multiple measures at either baseline or/and follow up. LAMP3, with the highest concentration at baseline demonstrated a weak to moderate negative correlation to FVC% at baseline and follow up, while also being weakly positively correlated to CPI at both time points. MCP-3 showed a similar pattern at baseline and follow up with a negative correlation with FVC % and TLC %, in addition to a positive correlation with CPI at follow up. Likewise, MMP7 showed a weak-moderate negative correlation to FVC % and TLC % at baseline and follow up, while a positive association with CPI at follow up was observed as well. Albeit weakly, but positively correlated with DLCO % at baseline was DCN, an association that was absent at follow up. However, DCN was negatively correlated with CPI at both baseline and follow up and showed a positive correlation with FVC % at baseline. The remodeling associated protein HGF, which was increased in IPF patients, exhibited negative correlations to FVC %, TLC% and CPI at baseline, but only TLC % at follow up. 

Other proteins that rendered associations to several measures were, e.g., ARG-1, which was abundantly expressed in IPF patients compared to controls. While no associations with lung function or CPI was observed at baseline, ARG-1 demonstrated an inverse correlation with all lung function measures; FVC %, TLC % and DLCO %, and a positive correlation with CPI at follow up. An opposite but similar pattern was observed for PDGF-subunit B, which was not differentially expressed compared to controls and only associated to FVC % at baseline. At follow up however, PDGF-subunit B correlated with FVC %, TLC %, DLCO % and CPI.

**Figure 4:**
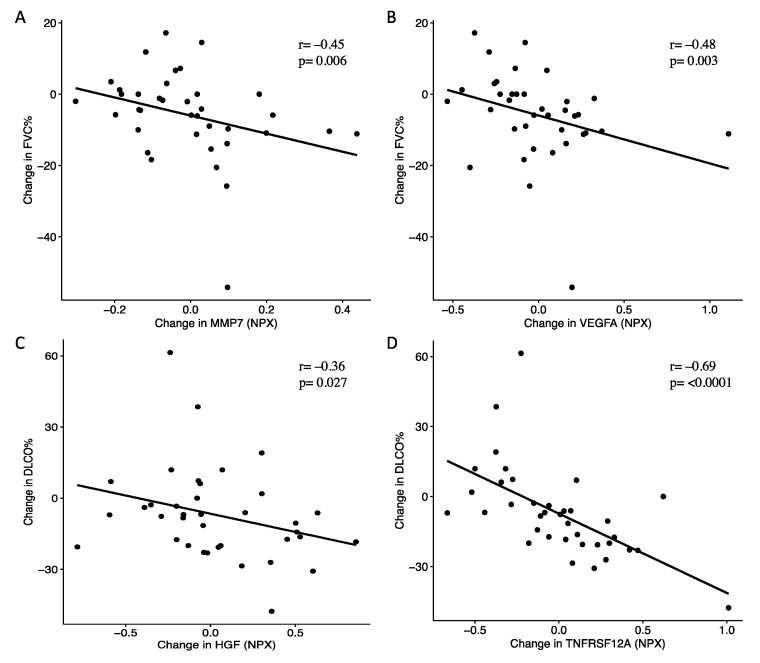
Elevated levels of MMP7, VEGFA, HGF and TNFRSF12A were associated with declining lung function. Increasing MMP7 and VEGFA correlated with decline in forced vital capacity (FVC %) (**A**,**B**), while HGF and TNFRSF12A were associated with decline in diffusing capacity (DLCO %) (**C**,**D**). *p* and r values were determined using Spearman rank correlation method.

We further investigated associations between longitudinal changes in protein levels and lung function (Table 6). In agreement with the associations to severity at baseline, changes in levels of proteins linked to remodeling and MMP7 in particular, also correlated with decline in lung function. Elevated levels of MMP7 were associated with decline in FVC % and DLCO %. Similarly, elevations of VEGFA and NOS3 correlated with declining FVC % and DLCO %. Additional proteins of remodeling that followed the change in lung function were PDGF-subunit B, HGF and MMP12, of which increasing levels of PDGF-subunit B were associated with decline in FVC %, and HGF and MMP12 with declining DLCO % (Figure 4). Changes in CXCL9 and IL12, proteins associated with inflammation/chemotaxis, were positively correlated with both changes in FVC % and TLC %. Albeit no correlations between the protein levels of TNFRSF12A and measures of lung function were observed at baseline, a strong negative correlation was observed between increasing levels in TNFRSF12A and changes in DLCO % (Figure 4). 

### 2.6. Comparison between Stable and Progressive IPF Patients 

Next, patients were divided into stable and progressive disease. Twenty patients had progressed while 17 patients remained stable at follow up. Patients with progressive disease had higher levels of IL6, NOS3, MMP7 and CASP-8 at follow up (mean difference 0.45 (95% CI of difference: 0.03–0.88), *p* = 0.04; 0.51 (95% CI: 0.02–1.01), *p* = 0.03; 0.14 (95% CI: 0.02–0.25), *p* = 0.03 and 0.36 (95% CI: −0.01–0.73), *p* = 0.03, respectively). Despite not reaching statistical significance when comparing the two groups, levels of ARG1 at follow up were numerically increased in progressive patients (mean difference 0.47 (95% CI: −0.08–1.02), *p* = 0.05). CXCL13 levels in progressive patients at follow up were also numerically increased compared to stable patients but not statistically significant (0.43 (95% CI: −0.07–0.93), *p* = 0.17). IL12 demonstrated an opposite pattern, where progressive patients had numerically lower, but not statistically significant levels at follow up compared to stable patients (−0.60 (95% CI: −1.13–0.07), *p* = 0.09). 

We further investigated changes in protein levels between baseline and follow up in patients with progressive and stable disease (Table S5). Patients with progressive disease demonstrated, compared to stable patients, increasing levels of several proteins associated with tissue remodeling such as NOS3 (progressive: mean difference 0.44 (95% CI of difference: 0.002–0.89); stable: −0.04 (−0.21–0.12), *p* = 0.01)), HGF (0.14 (95% CI: −0.04–0.33); stable: −0.1(95% CI: −0.25–0.05), *p* = 0.04)), VEGFA (0.08 (95%CI: −0.07–0.23); stable: −0.12 (95% CI: −0.24- (−0.0001)), *p* = 0.03)), CASP-8 (−0.035 (95% CI: −0.31–0.24); stable: −0.49 (−0.79- (−0.19)), *p* = 0.02) and MMP7 (0.04 (95% CI: −0.03–0.12); stable: −0.06 (−0.13–0.005), *p* = 0.03)). Furthermore, levels of TNFRSF12A (0.15 (95% CI: 0.007–0.30); stable: −0.19 (−0.35- (−0.04)), *p* = 0.0008)) increased during the observation period in progressive patients while it decreased in patients who were stable.

**Figure 5:**
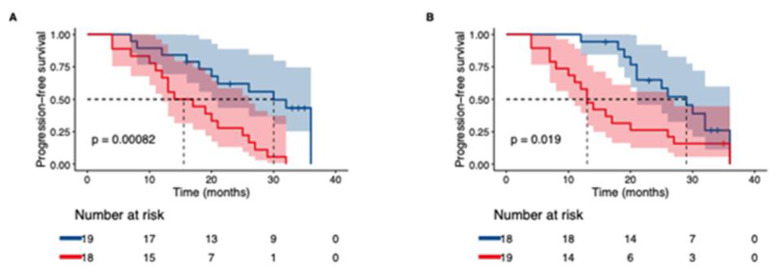
Kaplan–Meier curves for time to first IPF-progression (defined as ≥ 10% relative decline in FVC% or ≥15% decline in DLCO%). Lung function tests over a 36 months period from baseline were considered. Groups are stratified by protein change (elevation/reduction) observed at follow up. For each biomarker, red indicates the group of patients with elevated levels of respective protein observed at follow up. Blue indicates the group of patients with decreasing levels of protein observed at follow up. (**A**) MMP7, (**B**) TNFRSF12A.

Based on the results obtained above (Table S5), we proceeded with Kaplan–Meier analysis to explore if patients with elevations in MMP7, TNFRSF12A, NOS3, HGF, VEGFA and CASP-8 progressed faster than patients who showed the opposite pattern. Compared to the analysis on progression presented above, this analysis takes into consideration all lung function tests performed by the patients over a 36-month period, whilst the previous analysis only considered the lung function tests made in connection with baseline and follow up sampling. Patients with increasing levels of MMP7, TNFRSF12A, NOS3, CASP-8 and TWEAK at follow up demonstrated a faster rate to progression compared to patients with reduced levels (Figure 5A,B and Figure S3A–D.

In the univariate Cox proportional hazards regression analyses (Table S6) of baseline levels of the proteins deemed significant in the progression analyses, including CD40 due to its significance in the ex vivo model, CXCL13 for its established association to progression observed in the literature, and TWEAK due to its connection with TNFRSF12A, only TNFRSF12A yielded a significantly decreased hazard ratio for progression (HR: 0.28, 95% CI: 0.09–0.99, *p* = 0.047). Following adjustments for age, gender and baseline FVC % and DLCO %, baseline values of both TNFRSF12A (HR:0.18, 95% CI: 0.04–0.77) and its ligand TWEAK (HR:0.13, 95% CI: 0.02–0.74) were predictive of non-progression. In contrast, multivariate Cox regression analyses of the change in each respective biomarker revealed that patients with increasing levels of MMP7 (HR: 63, 95% CI: 4.36–918, *p* = 0.002), TNFRSF12A (HR: 3.3, 95% CI: 1.24–8.92, *p* = 0.02), TWEAK (HR: 7.9, 95% CI: 1.71–36.2, *p* = 0.008), VEGFA (HR: 4.2, 95% CI: 1.27–13.8, *p* = 0.02), NOS3 (HR: 1.7, 95% CI: 1.12–2.58, *p* = 0.01), CD40 (HR: 9.24, 95% CI: 1.95–43.8, *p* = 0.005) and CXCL13 (HR: 2.0, 95% CI: 1.03–3.88, *p* = 0.04) progressed faster than patients with decreased levels.

### 2.7. Outcome of Treatment Effects in Protein Expression 

We also analyzed the protein levels in serum from IPF patients with regards to treatment with antifibrotics. Patients who had initiated treatment between baseline and follow up (n = 13) had a change in level of 30 proteins as opposed to 8 proteins in patients treated throughout the observation period (n = 12). In patients not treated with antifibrotics (n = 11), only TRAIL was changed during the period (Table 7). Patients who had initiated treatment demonstrated a clear decrease in the expression of the remodeling proteins EGF, FGF2 and VEGFR-2 (Table 7). Concurrent with the reduction in proteins of remodeling, several proteins involved in inflammation such as TNFSF14, CD40-L, CCL3, IL-6 and the protein CASP-8 with overlapping functions, also demonstrated decreased levels at follow up. Decreasing levels in the remodeling protein FGF2 were observed in patients who were treated at both baseline and follow up as well. Meanwhile, protein levels of PTN, GZMB, MMP12 and CD8a increased in the same group. In terms of progression of the disease in each treatment group, no differences were observed.

## 3. Discussion

Experimentally induced models of lung fibrosis such as the bleomycin model are commonly used to study fibrotic development and therapeutic interventions in vivo [23,24] however, showing difficulties in recapitulating human features and mirroring clinical outcomes. By culturing healthy lung fibroblasts, a main player in ECM production and regulation, in a distorted lung environment that is naturally developed in patients with IPF, we can monitor the cellular responses activated by the intrinsic properties of the fibrotic tissue, which enables the development of new targets. In this exploratory translational study, we use a novel human ex vivo model to examine the intricate pulmonary interplay between cells, i.e., fibroblasts and ECM in IPF. Most importantly, results were validated in serum from a well characterized cohort of IPF patients. The results add further evidence and understanding for the mechanistic complexity of the disease by the sustained upregulation of several proteins involved in tissue remodeling, inflammation and chemotaxis, and the associations of these proteins to disease physiology, severity and progression. 

### 3.1. Inherent ECM Properties Trigger Fibroblast Activation

Ten out of the 12 proteins identified as significantly elevated in repopulated IPF scaffolds were also significantly elevated in serum from IPF patients, demonstrating strong cell-ECM crosstalk and translatability of the ex vivo as a pre-clinical tool. The two proteins from the ex vivo model that were not found to be elevated at serum baseline, TNFRSF21 and DCN, were however significantly elevated in serum samples that were unadjusted for age (Table S1). Interestingly, we found that the protein pattern between baseline and follow up samples in IPF patients differed as the amount of significantly elevated inflammatory/chemotaxis mediators in IPF were reduced at follow up (30 proteins at baseline vs 18 proteins at follow up), while the expression of remodeling proteins was more consistent (7 proteins at baseline vs 9 proteins at follow up). This shift in mediators was also reflected in our ex vivo model between day 1 and day 9, suggesting a temporal activation and de-activation of biological processes in the disease that may in part be reflected ex vivo. 

### 3.2. Remodeling Processes Linked to Inflammatory Processes

IPF is thought to be a result of repeated micro-injuries in the lung epithelium creating an abnormal and ongoing wound repair that initiates an inflammatory response. The massive recruitment and infiltration of inflammatory cells such as monocytes [25,26], and fibrocytes [27] is followed by a remodeling phase with fibroblast activation and myofibroblast differentiation where the lung tissue becomes re-organized. The extensively pathologically remodeled lung creates a stiffer and less compliant tissue, which has a major impact on local cellular behavior [16,17]. We found that fibroblasts exposed to fibrotic IPF-matrix responded by secreting increased amounts of MMPs, DCN and PGF, all of which are involved in processes of ECM remodeling [28,29,30,31,32]. Equally at baseline, serum samples from IPF patients demonstrated increased levels of proteins regulating tissue remodeling, of which the majority influences epithelial and vascular development such as HGF, MMP7, MMP12, VEGFA, ANGPT2 and PGF [33,34,35]. Both MMP7 and MMP12 aid in the reconstruction and maintenance of lung tissue through the proteolytic cleavage of ECM components and basement membranes (BM) as well as ECM-bound growth factors, causing a local release of matrikines and signaling molecules that can create a chemotactic gradient reinforcing local cellular infiltration of mesenchymal and inflammatory cells, as well as their activation and differentiation [36,37,38]. MMP7, described as a potential diagnostic and prognostic biomarker of IPF [39,40] specifically cleaves DCN [41], a proteoglycan involved in the fibrillization of collagens and acts as an inhibitor of the powerful profibrotic agent transforming growth factor (TGF)-β that induces myofibroblast differentiation [42,43,44]. This influential aspect of MMP7 on TGF-β may aid in maintaining a fibrotic active status. Though DCN was not found to be elevated in IPF serum after age-adjustment, the increased MMP expression in IPF providing an elevated ECM turnover may be mirrored in the systemic circulation, suggesting that the elevated serum levels of fragmented DCN in IPF patients might be due to the disease and not age [45]. The enzymatic breakdown and re-build of the ECM is a prominent pulmonary feature in IPF and particularly affect the BM, as shown in our previous study [13]. A fragmented BM was shown in IPF lung, with altered production of BM-associated proteins such as collagen VI and collagen IV, the latter a collagen targeted by MMP7 and MMP12 [28,46]. Emerging evidence also point toward endothelial dysfunction and structural alteration of the underlying BM, which may contribute to the interstitial pulmonary fibrosis seen in post-COVID-19 patients [47,48].

### 3.3. Inflammatory Processes Linked to Remodeling

Several proteins engaged in inflammation and chemotaxis were elevated by fibroblasts cultured in IPF scaffolds, of which CXCL13 and CCL19 share a close connection in the recruitment of B cells and T cells [49,50]. Intriguingly, both CXCL13, demonstrated as a promising prognostic biomarker for IPF [51], and CCL19 were found amongst the top four proteins most elevated in IPF serum at baseline. One feature seen in IPF patients is the presence of lymphoid follicles in the lung, containing nonproliferating activated B cells, T cells and dendritic cells [52]. These structures, initiated by local recruitment of T cells and B cells are closely situated near blood vessels that selectively express T cell attracting chemokines. Fibroblasts, prone to regulate the infiltration and accumulation of inflammatory cells, may suggest that the fibrotic microenvironment in IPF creates an ECM-driven fibroblastic phenotype with more invasive and pro-inflammatory characteristics. In a murine lung metastasic model, CCL19 expressing fibroblastic stromal cells promoted the accumulation of CD8+ T cells [50], which correlated with an increased gene expression of CCL19 in these cells in human lung tumors, further emphasizing the immune modulatory impact of fibroblasts. 

When examining the protein interaction of all elevated mediators in IPF, we found a distinct cluster of proteins regulating inflammation and chemotaxis such as CXCL12, which is involved in fibrocyte homing to the lung [53]. The cluster was mainly composed of CXC-chemokines that have been recognized as regulators of vascular development, with angiogenic and angiostatic properties [54]. The remodeling proteins, also identified to be elevated in the ex vivo model, were found to be closely linked to HGF, IL-6, TNF and VEGFA. VEGFA acts as a powerful angiogenic mediator and it has been described that IL-6 can induce the expression of VEGF [55] as seen in synovial fibroblasts from RA patients [55]. We have shown that fibroblasts in IPF lung become activated in synthesizing increased amounts of collagens to uphold a fibrotic tissue with typical IPF features, of which collagen I has been described to act as a scaffold for angiogenesis [56]. The mixture of chemotactic agents for cellular recruitment and vascular formation creates chemokine-guided angiogenesis that designs a local pulmonary niche that constantly pushes ECM turnover, creating a permanent and chronic state of tissue remodeling. 

### 3.4. Protein Association to Disease Severity and Progression

With our longitudinal collection of samples, we are able to study if associations to common measures of disease severity are maintained over time, providing further evidence for the role of both established proteins and candidates in IPF. Taken together, our data demonstrate the significance of several proteins involved in remodeling, of which MMP7 was indicative of being the most prominent across our analyses. Other reoccurring remodeling proteins with regards to associations to severity and progression were HGF, VEGFA, MMP12, NOS3 and TNFRSF12A. The elevated levels of HGF and its correlations to disease severity and progression observed in this study correspond with other studies of fibrotic lung diseases [57,58,59,60,61]. Known for being involved in repair and regeneration of injured lung tissue [62], in vivo and in vitro studies have pointed out HGF as a protein with antifibrotic effects [63,64,65,66], while others have speculated that increased HGF levels are a result of lung injury [60]. Our results showing both elevations and associations to disease severity might be in support of the latter. 

As the fibrotic destruction of the alveoli in IPF ultimately leads to a reduced gas diffusion capacity over time, the strong association observed between changes in TNFRSF12A and DLCO % is interesting. TNFRS12A is a member of the TNF receptor superfamily, which has been described to act as a downstream target of TGF-β and to contribute to pathological angiogenesis through the binding of the multifunctional cytokine TWEAK [67,68]. In fibrotic kidney disease the TWEAK-TNFRS12A signaling pathway has been identified as important in the activation of myofibroblasts, where TWEAK was shown to regulate the gene expression of leukocyte recruiting chemokines such as CXCL1, CXCL10 and MCP-3 and the release of proinflammatory cytokines IL-6 and MCP-1 in myofibroblasts [69]. Of interest, TWEAK was found to be negatively correlated to FVC %, DLCO % and CPI, further implicating the activation of TNFRSF12A by TWEAK as a possible fibrogenic pathway in the lung. Further, our data underline the important role of inflammation and chemotaxis in the pathophysiology of IPF. For example, the increased levels of monocyte chemoattractant proteins (MCP-1, MCP-3, MCP-4) in both the age-adjusted and non-adjusted analyses, with special emphasis on MCP-3 with its correlations to several measures of disease severity, demonstrate and contribute to the literature their involvement in the development of fibrosis [70,71,72,73]. TNFSF14 (LIGHT) is another interesting example of an inflammatory protein, which was increased in IPF patients and correlated to several measures of severity. TNFSF14 exerts its effects on, e.g., epithelial cells, T-cells, macrophages and fibroblasts, and can regulate their accumulation, maintain an inflammatory milieu by upregulating adhesion proteins but also promote collagen deposition and remodeling [74,75,76]. Intriguingly, TNFSF14′s role in the initiation and perpetuation of fibrosis has been shown in several other inflammatory diseases with a fibrotic element, such as liver [77], skin [78] and renal [79] fibrosis.

Of relevance, MCP-3 has been found in several studies in blood from patients hospitalized with COVID-19 infection [80,81,82]. In these studies, MCP-3 has been shown to correlate with the severity of COVID-19 infection with elevated levels observed in patients with severe disease, further supporting the role of monocytes in the pathogenesis. Also of interest is that sustained upregulations of HGF, TNFSF14 and CASP-8 have been observed in studies on COVID-19 patients [80,82] as an indicator for severe disease. 

### 3.5. Progressors vs. Stable Patients

The results on how protein levels are altered with regard to progression/non-progression should only be seen as hypothesis generating given the low numbers of patients. However, our findings show that progressive disease has higher levels of IL6, NOS3, MMP7 and CASP-8 compared to stable patients at follow-up. Albeit not reaching statistical significance, levels of ARG1 and CXCL13 were numerically higher in patients who had progressed at follow up, while levels of IL-12 were numerically lower in progressors. In parallel, progressive patients also demonstrated increasing levels of among others, NOS3, VEGFA, HGF, MMP7 and TNFRSF12A, further emphasizing several ongoing vascular and tissue remodeling processes linked to progression of the disease. Adding results from the Kaplan–Meier and cox-regression analyses demonstrate that the progression of disease involves multiple pathways. The analysis of the predictive value of baseline TWEAK and TNFRSF12 and their “protective” effects against progression comes off as paradoxical due to their associations to disease severity and the subsequent analyses of how patients with elevations in these proteins show a faster rate to progression. Prospective studies are certainly needed to confirm these results and evaluate the role of TWEAK and TNFRSF12A in fibrosis and disease development.

### 3.6. Treatment Effects

We provide explorative data on the potential effects of antifibrotic treatments on protein levels. In patients who were untreated at baseline but treated at follow up and thus the group that perhaps best can convey the direct outcome of treatment, a reduced effect was demonstrated both on remodeling associated proteins (e.g., EGF, FGF2, VEGFR-2 and TIE2), and inflammatory proteins (e.g., TNFSF14, CD40-L, CD40, CCL3 and IL-6). From a pharmacodynamic perspective, the decrease in tyrosine kinase associated proteins are in line with the mechanisms of which the approved antifibrotic treatments exert their effects [5,83,84]. Moreover, both nintedanib and pirfenidone have been shown to exhibit anti-inflammatory properties in vitro [83,85]. However, we did not observe any differences between treatment groups in regard to the number of patients progressing or not. Together with the similarities seen in the levels from the cohort of patients with treatment at both baseline and follow up, with decreasing levels of, e.g., FGF2, CD28 and CD244, our results propose specific proteins and biological pathways modulated by treatment exposure. 

As pointed out in several biomarker-related studies over the years, a “one-protein” approach to establish diagnosis or prognosis is likely not feasible given the large number of active pathophysiological processes in IPF. Rather, an evaluation of several markers reflecting different pathophysiological events may be more relevant. A main limitation of our study is the relatively small cohort sizes, which limits the power in our analyses and likely overestimates the risks reflected by the hazard ratio and confidence intervals in the cox-regression analyses. The absence of a replication cohort to validate our results and the potential influence of antifibrotic treatments, immunomodulatory agents such as corticosteroids, comorbidities or other unmeasured variables on the expression of analyzed biomarkers are also factors that ought to be weighted in when interpreting our results. Despite this, using our human ex vivo model, we have identified markers of remodeling, inflammation and chemotaxis that are differentially expressed compared to controls, and are associated with disease severity. We also demonstrate how changes in protein levels may track with changes in lung function. These types of longitudinal measures are relatively unique among studies of biomarkers in clinical studies of IPF patients. By combining clinical data with biological markers evaluated in experimental human models we believe that more accurate information will be achieved which, in turn, may increase our understanding of the pathobiology of IPF. Our data suggest that remodeling events linked to inflammatory processes governed by the cell-ECM interplay are the main drivers of the disease.

## 4. Materials and Methods

### 4.1. Study Design

This study was designed in two parts; firstly, to examine local fibroblast activities in IPF using a novel ex vivo model, and secondly to compare and establish the systemic proteomic profile in serum collected at baseline and follow up from IPF patients. By linking the proteomic data to clinical data such as lung function, we sought to find markers indicative of disease severity and progression. 

### 4.2. Ex Vivo Model 

Human distal lung tissue was dissected from healthy donor lungs (resection or explanted material), or explanted lungs from IPF patients that were diagnosed consistent with ERS and ATS criteria (Raghu et al., 2018). Native lung samples were cut into 1 cm^3^ blocks and directly frozen in 2-methylbutan chilled with liquid nitrogen. Lung tissue originated from four IPF patients; two females (57 years and 62 years old) and two males (57 years and 68 years old) with prior smoking history with forced vital capacity (FVC) ranging between 1.1–3.1 L. Healthy donor tissue was retrieved from two females (41 years and 55 years old) and two males (62 years and 86 years old) of which three were non-smokers and one a current smoker. Lung tissue was cryosectioned into 350 µm slices and treated for cellular removal according to previously established decellularization procedure [13,86]. In short, lung tissue slices were washed repeatedly with detergent solution CHAPS and treated with benzonase nuclease to generate acellular lung slices with intact ECM composition and structure (scaffolds). 

**Figure 6:**
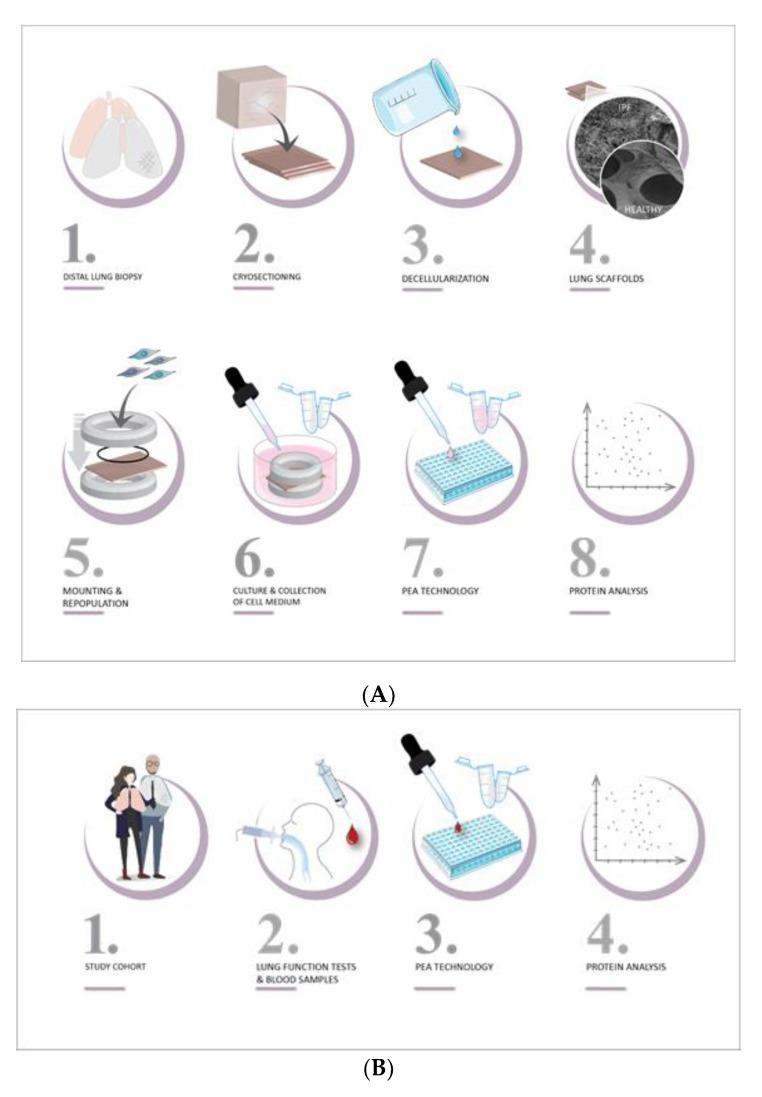
Translational study design of protein profiling of IPF. (**A**) top image, (**B**) bottom image. (**A**) Ex vivo model. (1) Cubes of 1 cm^3^ of distal lung tissue were dissected from lungs of healthy donors and IPF patients, and directly frozen. (2) Tissue was cryosectioned into 350 µm thin slices. (3) Cells were removed with detergent solution containing CHAPS. (4) The decellularized lung tissue (scaffolds) presented maintained tissue morphology with disease features. (5) Scaffolds were repopulated with healthy lung fibroblasts and mounted in custom-made holders, keeping scaffolds in a stretch formation. (6) Repopulated scaffolds were cultured up to 9 days, where cell medium was collected at day 1 and day 9. (7) 92 proteins (immuno-oncology panel) were analyzed in collected cell medium using proximity extension assay technology. (8) The proteomic profile of each sample was reported as relative protein amount (NPX value). (**B**) IPF patients. (1) A cohort of 77 healthy donors (smokers and non-smokers) and 38 IPF patients (from the IPF-registry) were included. (2) Blood samples from IPF patients were collected at baseline and at follow up with associated lung function parameters and clinical data. (3) Serum samples were analyzed for 92 proteins (immuno-oncology panel) using PEA technology. (4) The proteomic profile of each sample was detected reported as NPX value and correlated to lung function parameters.

Healthy distal lung fibroblasts, derived from one donor, were used to repopulate scaffolds placed in 24-well suspension plates with mild agitation at 10% CO_2_ at 37 °C. After 24 h, lung scaffolds derived from healthy donors or IPF patients were mounted on custom-made holders (8 mm inner diameter) to maintain lung tissue in a stretch formation, as fibroblast overtime contract surrounding tissue. Repopulated scaffolds were cultured up to 9 days in complete SILAC DMEM Flex Media (Life Technologies, Carlsbad, CA, USA, cat.no. A2493901) supplemented with 10% dialyzed serum (Gibco, A3382001), glucose (4500 µg/mL), amphotericin B (2.5 µg/mL), penicillin-streptomycin (1%), gentamicin (50 µg/mL), 1% Glutamax, with medium changed at day 1, 3 and 6. For detailed methodology, please see Figure 6A and the method section in our previously reported study [13]. Cell culture media were collected after 1 day and 9 days in culture and analyzed for protein content. 

### 4.3. Serum from IPF Patients and Controls 

We used serum from patients included in the Swedish IPF-registry [87,88] that enrolls both prevalent and incident cases (Figure 6B). The IPF cohort of this study consisted of 38 patients enrolled from Karolinska University Hospital, Solna, Stockholm, Sweden, all with a confirmed diagnosis of IPF according to national [89] and international guidelines [1,90,91]. Serum samples were collected, aliquoted and stored at −70 °C within two hours of sampling. No exclusion criteria were applied based on age, gender, comorbidities or concomitant medication, including antifibrotic treatments.

Two serum samples from each patient were obtained: at baseline and upon follow-up. The baseline samples (n = 38) were taken at a median time of 2 months (IQR: 11.8 months) from diagnosis. Follow-up samples were obtained at time points ranging from 6–30 months (median: 16 months (IQR: 9.5 months)) from baseline. Thirty-seven patients were included in the follow-up analyses as one sample did not meet the quality control guidelines set for protein analysis. Associated clinical data included forced vital capacity, % predicted (FVC %), diffusing capacity for carbon monoxide, % predicted (DLco %) and total lung capacity, % predicted (TLC %). Four measures of disease severity were considered: FVC %, DLco %, TLC %, and the composite physiologic index (CPI), which correlates with the fibrotic extent seen in radiology [92]. Disease progression during the follow-up period was defined as a decline of at least 10% in FVC % and/or a decline of at least 15% in DLco % between the baseline lung function and the lung function at follow-up. The data were taken from procedures conducted maximum 6 months before or after collection of the serum samples. 

Serum from the COpd and Smoking from an oMIC perspective (COSMIC) cohort conducted at Karolinska Institutet and Karolinska University Hospital, Stockholm, Sweden (NCT02627872) was used as a healthy control cohort [93,94,95]. One serum sample was collected from 37 never smokers and 40 current smokers with normal lung function. The controls reported no history of allergy or asthma nor any use of either oral or inhaled corticosteroids. 

### 4.4. Protein Analysis 

A panel of 92 proteins were analyzed both in cell culture media from the ex vivo model (Figure 6A) and in human serum samples from IPF patients and controls (Figure 6B). The immune-oncology panel by Olink Proteomics AB (Uppsala, Sweden) uses proximity extension assay (PEA) technology to identify and quantify proteins using two oligonucleotide-conjugated antibodies that bind to each specific protein target [96]. Upon binding to the protein, the oligonucleotide pairs are amplified through quantitative PCR (qPCR). Protein concentrations are expressed using Normalized Protein eXpression (NPX) values on a log2 scale, where a high NPX value represents a high protein expression. In analyzed serum samples and in cell medium samples, proteins with detectability lower than 67% (>33% of limit of detection per protein) were excluded, as well as samples failing quality parameters set for the analysis with inter- and intra assay variability controls. The final protein panel to be evaluated included 87 (95%) and 41 (45%) proteins out of the 92 examined proteins in serum samples and cell medium, respectively. Proteins were categorized into two main categories, inflammation/chemotaxis and tissue remodeling according to annotated biological functions reported in bioinformatic databases; the Uniprot knowledgebase and Gene Ontology Proteins where distinct overlapping functions were categorized accordingly.

Bioinformatic analysis of protein interactions was analyzed and illustrated with STRING database v 11, showing full network with line thickness denoting confidence strength and textmining, experiments, databases and co-expression as active interaction sources, illustrated with Cytoscape v.3.8.2. Note that the following proteins are shown with corresponding gene identification in STRING; ADGRG1 = GPR56, MCP-3 = CCL7, MCP-4 = CCL13, Gal-9 = LGALS9, Gal-1 = LGALS1 and PD-L1 = CD274. Pathway analysis was performed with g:Profiler with ranked gene list according to mean NPX difference using KEGG and Reactome databases for biological function annotations. 

### 4.5. Statistical Analyses

Two-way ANOVA with Sidak’s adjustment for multiple comparison was used for the identification of differentially expressed proteins between repopulated healthy and IPF scaffolds in the ex vivo model. Serum samples were analyzed with one way ANOVA for comparisons of IPF patients vs. controls. As the mean age of the IPF cohort is 18.2 years older than the healthy cohort, the ANOVA was adjusted for the age discrepancy only to avoid overfitting. Protein selections were corrected for multiple testing using the Benjamini–Hochberg method, controlling for a false discovery rate (FDR) at 5%. Differences between groups were assessed using Mann–Whitney test, while paired data was analyzed with Wilcoxon signed rank test. Correlation analyses between protein expression and clinical variables were done using Spearman’s rank correlation. Analyses of time to progression were performed using Kaplan–Meier curves to illustrate the associations of changes in protein level (stratified by increase or decrease in levels observed at follow up) with progression. Progression-free survival was defined as the time from baseline until the date of first disease progression. Comparisons were done using the log-rank test. Patients were censored at the time of the last spirometry performed during the observation period of maximum 36 months from baseline. Univariate and multivariate Cox regression analyses were used to investigate the associations between continuous levels of proteins deemed important in progression and drop of either >10% in FVC% or >15% in DLCO% over a 36-month period. The covariates considered in the multivariate analysis included age, gender, FVC% and DLCO% at baseline. Results were considered significant if the *p*-value was <0.05. Even though the statistical tests have been adjusted for covariates and post hoc comparisons to avoid erroneous significance levels the study employs multiple hypothesis testing, where each hypothesis was analyzed separately and the existence of patterns in and the consistency of the results were considered in the analysis. Statistical analyses were performed in SAS (The SAS system for Windows 9.4., SAS Institute INC., Cary, NC, USA), GraphPad Prism 9 and R (R Core Team, 2020, version 3.6.3). Continuous variables are reported as means with SD. Categorical variables are presented as counts and percentages. Bioinformatical analysis with q:Profiler was performed on all known genes as statistical domain scope with over-representation analysis with Bonferroni correction with significant threshold of 0.01.

## 5. Conclusions

**Figure 7:**
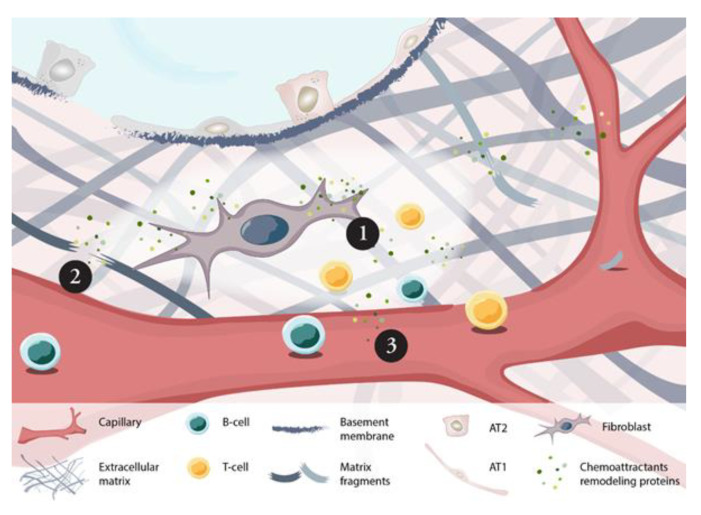
Proposed mechanism of local fibroblast activity in IPF lung revealed in patient serum. In distal lung tissue of IPF patients, fibroblasts become activated by the surrounding fibrotic-ECM triggering a profibrotic phenotype releasing elevated levels of chemoattractants (e.g., CCL19, CXCL13) for the recruitment of immune cells (1), and of remodeling proteins (e.g., MMP7) (2). The released factors alter ECM turnover, influencing the integrity of epithelial and vascular basement membranes facilitating and cellular invasiveness and responses in the tissue. Fibroblast-ECM interplay renders a systemic fingerprint in serum of IPF patients (3) that correlate to disease severity and ongoing local tissue remodeling processes in the lung.

In conclusion, we have explored proteins related to remodeling of the ECM, inflammation and chemotaxis both ex vivo and in serum from patients with IPF. We emphasized the crosstalk between fibroblasts and ECM, revealed in our preclinical experiments, as an important interplay in IPF as their local interaction in part was reflected in the systemic profile of IPF patients including correlation to disease severity. The constant release of recruitment factors by fibroblasts in a fibrotic environment creates a persistent homing of inflammatory cells such as monocytes, T cells and B cells to the site of injury where lack of intact barriers in epithelial and vascular BM, created by the activity of, e.g., MMPs, further enables cellular infiltration and chronic tissue injury (Figure 7). By correlating the proteomic data to measures of disease severity and progression, we have been able to validate established markers of diagnostic and prognostic value while also proposing other novel biomarker candidates such as MMP7, VEGFA, TNFRSF12A, HGF and MCP-3.

## Figures and Tables

**Table 1:** Proteins significantly elevated in cell medium from repopulated IPF scaffolds. In comparison to healthy scaffolds (HL), fibroblasts cultured on IPF scaffolds demonstrated an increased release of 12 proteins. Table show proteins ranked based on difference in group mean (NPX values) between IPF and healthy condition, categorized into two main biological functions. Two-way ANOVA with Sidak’s multiple comparison test, * *p* < 0.05.

Protein	Mean IPF Day 1 log2FC ± SD	Mean HL Day 1 log2FC ± (SD)	Mean Difference (IPF−HL) Day 1	*p*−Value (Day 1)	Mean IPF Day 9 log2FC ± SD	Mean HL Day 9 log2FC ± (SD)	Mean Difference (IPF−HL) Day 9	*p*−Value (Day 9)
MMP7	4.67 (2.01)	1.13 (1.00)	3.54 *	5.7 × 10^−3^	1.87 (1.28)	−0.03 (0.73)	1.89	1.3 × 10^−1^
MMP12	7.83 (0.61)	6.24 (0.62)	1.59 *	3.1 × 10^−2^	5.23 (0.56)	3.74 (1.22)	1.53 *	3.8 × 10^−2^
PGF	3.51 (0.60)	1.92 (0.30)	1.56 *	5.0 × 10^−4^	2.09 (0.18)	1.42 (0.54)	0.66	1.1 × 10^−1^
DCN	6.96 (0.40)	6.51 (0.36)	0.45	2.4 × 10^−1^	6.42 (0.46)	5.22 (0.33)	1.20 *	1.8 × 10^−3^
**Inflammation/Chemotaxis**								
CXCL13	9.94 (1.83)	3.59 (0.70)	6.35 *	1.0 × 10^−4^	8.01 (2.00)	2.62 (1.12)	5.38 *	6.0 × 10^−4^
GAL9	9.01 (0.93)	5.69 (0.56)	3.31 *	<1 × 10^−4^	5.84 (0.46)	3.01 (0.40)	2.84 *	<1 × 10^−4^
GZMA	2.57 (1.91)	0.10 (0.37)	2.48 *	8.9 × 10^−3^	−0.16 (0.43	−0.49 (0.23)	0.33	8.8 × 10^−1^
CD40	8.17 (1.49)	6.22 (1.58)	1.96	6.6 × 10^−2^	6.40 (0.45)	4.18 (0.61)	2.22 *	3.6 × 10^−2^
CCL19	3.96 (1.05)	2.24 (0.71)	1.73 *	2.7 × 10^−2^	2.45 (0.87)	1.12 (0.71)	1.35	8.6 × 10^−2^
CD4	1.85 (1.14)	0.25 (0.76)	1.60 *	1.6 × 10^−2^	−0.26 (0.14)	−1.01 (0.38)	0.74	3.0 × 10^−1^
TNFRSF9	1.35 (0.60)	−0.20 (0.41)	1.55 *	2.0 × 10^−4^	−0.04 (0.21)	−0.71 (0.19)	0.68	6.0 × 10^−2^
TNFRSF21	2.09 (0.33)	1.56 (0.09)	0.52 *	7.6 × 10^−3^	1.36 (0.15)	1.33 (0.20)	0.03	9.7 × 10^−1^

**Table 2:** Characteristics of IPF patients and controls.

	IPF (*n* = 38)	Controls (*n* = 77)
Age (Mean ± SD)	73.8 ±7.83	55.6 ± 6.7
Male/Female, n (%)	29/9 (76%/24%)	39/38 (51%/49%)
Smoking history		
- Never smokers (n, %)	8 (21%)	37 (48%)
- Ex-smokers (n, %)	29 (76%)	-
- Current smokers (n, %)	1 (3%)	40 (52%)
Lung function		
- FVC (% predicted)	80.8 ± 20.2	113 ± 14.4
- FEV_1_ (% predicted)	81.2 ± 17.9	103 ± 13.9
- DLCO (% predicted)	50.4 ± 11.8	86.5 ± 14.0
- TLC (% predicted)	64.3 ± 11.2	107 ± 11.0
CPI (Mean ± SD)	43.0 ± 10.7	NA
GAP stage (n, %)		NA
1	21 (55%)	
2	17 (45%)	
3	0 (0%)	
Treatment with antifibrotics at serum sampling		NA
- Treated baseline and treated follow-up	12 (32%)	
- Untreated baseline and treated follow-up	13 (34%)	
- Untreated baseline and untreated follow-up	11 (29%)	
- Treated baseline and untreated follow-up	2 (5%)	

IPF: Idiopathic pulmonary fibrosis; FVC %:forced vital capacity, % predicted; FEV_1_ %: forced expiratory volume in 1 s, % predicted; DLCO %:diffusing capacity of carbon monoxide, % predicted; TLC %: total lung capacity,% predicted; CPI: composite physiological index; GAP-stage: gender-age-physiology stage for IPF.

**Table 3:** Elevated proteins at IPF baseline overlap with findings ex vivo. Proteins with differences in concentration (normalized protein expression, NPX) in baseline sample from IPF patients versus controls, categorized into two main biological function, tissue remodeling and inflammation/chemotaxis. Seven proteins were judged as having overlapping functions. ^a^ indicate proteins with differentiated expression observed in the ex vivo model. One-way ANOVA adjusted for age with Benjamini–Hochberg corrected *p*-value to control a false discovery rate at 5%.

Protein	NPX−Difference	*p*-Value	FDR Adjusted *p*-Value
Tissue remodeling			
ADGRG1	0.99	1.22 × 10^−5^	5.75 × 10^−3^
MMP12 ^a^	0.73	1.68 × 10^−4^	9.20 × 10^−3^
HGF	0.66	4.91 × 10^−8^	2.30 × 10^−3^
MMP7 ^a^	0.59	2.98 × 10^−1^1	1.15 × 10^−3^
VEGFA	0.54	1.13 × 10^−3^	1.38 × 10^−2^
ANGPT2	0.31	7.37 × 10^−3^	2.01 × 10^−2^
PGF ^a^	0.26	2.07 × 10^−4^	9.77 × 10^−3^
Inflammation/Chemotaxis			
LAMP3	1.34	2.47 × 10^−7^	3.45 × 10^−3^
MCP^−3^	1.33	1.01 × 10^−1^1	5.75 × 10^−4^
CCL19 ^a^	1.16	1.03 × 10^−7^	2.87 × 10^−3^
CXCL13 ^a^	1.00	4.31 × 10^−1^1	1.72 × 10^−3^
IL8	0.96	2.61 × 10^−6^	4.60 × 10^−3^
TNFSF14	0.93	4.84 × 10^−5^	7.47 × 10^−3^
CXCL11	0.75	4.51 × 10^−4^	1.15 × 10^−2^
CCL17	0.73	8.39 × 10^−4^	1.26 × 10^−2^
IL12	0.69	1.30 × 10^−3^	1.44 × 10^−2^
CXCL9	0.61	5.68 × 10^−3^	1.90 × 10^−2^
IL6	0.55	4.09 × 10^−3^	1.78 × 10^−2^
PDCD1	0.50	1.38 × 10^−4^	8.62 × 10^−3^
MUC^−1^6	0.46	1.10 × 10^−2^	2.30 × 10^−2^
CD83	0.46	1.81 × 10^−6^	4.02 × 10^−3^
PD−L1	0.44	3.51 × 10^−6^	5.17 × 10^−3^
MCP^−4^	0.43	1.34 × 10^−2^	2.41 × 10^−2^
CXCL1	0.43	3.24 × 10^−3^	1.72 × 10^−2^
TNFRSF4	0.42	6.91 × 10^−4^	1.21 × 10^−2^
IL18	0.42	1.55 × 10^−2^	2.47 × 10^−2^
LAG3	0.35	1.56 × 10^−2^	2.53 × 10^−2^
TNFRSF9 ^a^	0.35	2.90 × 10^−3^	1.67 × 10^−2^
CD28	0.32	2.43 × 10^−4^	1.09 × 10^−2^
CD27	0.31	1.79 × 10^−3^	1.55 × 10^−2^
CD4 ^a^	0.27	7.48 × 10^−5^	8.05 × 10^−3^
CD5	0.27	9.57 × 10^−3^	2.24 × 10^−2^
CD40 ^a^	0.26	4.61 × 10^−3^	1.84 × 10^−2^
Gal^−9 a^	0.23	1.27 × 10^−2^	2.36 × 10^−2^
CX3CL1	0.23	9.11 × 10^−3^	2.18 × 10^−2^
CSF^−1^	0.19	2.13 × 10^−3^	1.61 × 10^−2^
Gal^−1^	0.18	6.71 × 10^−3^	1.95 × 10^−2^
Overlapping functions			
ARG1	1.30	3.32 × 10^−5^	6.90 × 10^−3^
CXCL10	0.60	8.96 × 10^−3^	2.13 × 10^−2^
CCL23	0.49	2.27 × 10^−4^	1.03 × 10^−2^
ADA	0.48	2.56 × 10^−5^	6.32 × 10^−3^
TNF	0.34	1.08 × 10^−3^	1.32 × 10^−2^
TNFRSF12A	0.33	1.35 × 10^−3^	1.49 × 10^−2^
GZMA ^a^	0.31	7.92 × 10^−3^	2.07 × 10^−2^

**Table 4:** Temporal protein changes between IPF baseline and follow up. Protein levels (mean normalized protein expression, NPX) measured in serum from IPF patients that were changed between baseline and follow up. Proteins are categorized in two main biological functions, tissue remodeling and inflammation/chemotaxis. Three proteins have overlapping functions. Proteins significantly elevated in IPF compared to controls are indicated with ^b^. *p*-values calculated using Wilcoxon signed-rank test.

Protein	Baseline Mean NPX (±SD)	Follow Up Mean NPX (±SD)	Mean Difference (±SD)	*p*-Value
Tissue remodeling				
EGF	10.49 ± 0.84	10.08 ± 0.83	−0.39 ± 0.97	0.02
FGF2	0.93 ± 0.59	0.63 ± 0.55	−0.31 ± 0.49	0.0001
LAP TGF-beta-1	9.99 ± 0.37	9.81 ± 0.51	−0.16 ± 0.38	0.023
VEGFR-2	8.48 ± 0.22	8.35 ± 0.21	−0.12 ± 0.18	0.0002
HO-1	11.18 ± 0.40	11.07 ± 0.36	−0.1 ± 0.28	0.020
TIE2	7.70 ± 0.26	7.60 ± 0.24	−0.09 ± 0.16	0.0008
PTN	3.28 ± 1.38	3.57 ± 1.36	0.21 ± 0.59	0.046
Inflammation/Chemotaxis				
CCL3	6.62 ± 0.65	6.42 ± 0.50	−0.19 ± 0.54	0.042
CD28 ^b^	1.42 ± 0.31	1.27 ± 0.38	−0.15 ± 0.22	0.0002
CD244	6.26 ± 0.26	6.11 ± 0.31	−0.14 ± 0.2	0.0001
LAMP3 ^b^	6.85 ± 0.47	6.70 ± 0.46	−0.14 ± 0.39	0.015
IL12RB1	2.08 ± 0.30	1.96 ± 0.25	−0.12 ± 0.29	0.026
ADA ^b^	5.18 ± 0.36	5.06 ± 0.34	−0.11 ± 0.23	0.01
IL18 ^b^	8.96 ± 0.49	8.83 ± 0.45	−0.11 ± 0.29	0.027
MIC-A/B	4.62 ± 1.62	4.48 ± 1.60	−0.1 ± 0.21	0.009
CCL4	7.29 ± 0.54	7.20 ± 0.55	−0.09 ± 0.34	0.040
CD40 ^b^	10.03 ± 0.32	9.94 ± 0.35	−0.08 ± 0.25	0.033
ICOSLG	6.14 ± 0.19	6.07 ± 0.19	−0.07 ± 0.14	0.003
PD-L2	2.27 ± 0.25	2.23 ± 0.24	−0.05 ± 0.17	0.03
Overlapping functions				
CASP-8	4.36 ± 0.59	4.08 ± 0.57	−0.24 ± 0.62	0.02
TRAIL	8.57 ± 0.30	8.45 ± 0.32	−0.12 ± 0.2	0.002
TWEAK	9.13 ± 0.30	9.02 ± 0.33	−0.1 ± 0.26	0.01

**Table 5:** Protein levels in IPF serum correlated to disease severity. Correlations between protein levels in serum from IPF patients and disease severity, defined by forced vital capacity (FVC, % predicted), total lung capacity (TLC, % predicted), diffusion capacity for carbon monoxide (DLCO, % predicted) and composite physiological index (CPI). Proteins are categorized in two main biological functions, tissue remodeling (R) and inflammation/chemotaxis (I). Three proteins have overlapping functions (O). Proteins significantly elevated in IPF compared to controls are indicated with ^b^. Proteins observed in ex vivo model are indicated with ^a^. *p* and r values were determined using Spearman rank correlation method.

FVC %	Rho Coefficient	*p*-Value	Biological Function
MMP7 ^a,b^	−0.51	0.0006	R
HGF ^b^	−0.48	0.003	R
ANGPT1	−0.44	0.005	R
EGF	−0.43	0.007	R
LAP TGF-beta 1	−0.40	0.012	R
PDGF subunit B	−0.33	0.041	R
DCN	0.34	0.037	R
PTN	0.46	0.004	R
TNFSF14 ^b^	−0.54	0.0005	I
MCP-3 ^b^	−0.53	0.0006	I
LAMP3 ^b^	−0.46	0.004	I
CXCL1 ^b^	−0.43	0.008	I
CD40-L	−0.42	0.009	I
CXCL5	−0.33	0.042	I
MIC-A/B	−0.33	0.047	I
TWEAK	−0.35	0.03	O
MCP-1	−0.33	0.044	O
TLC %			
EGF	−0.52	0.0009	R
MMP7 ^a,b^	−0.47	0.003	R
ANGPT1	−0.47	0.003	R
HGF ^b^	−0.37	0.022	R
LAP TGF-beta 1	−0.34	0.040	R
PTN	0.33	0.042	R
TNFSF14 ^b^	−0.53	0.0007	I
CD40-L	−0.50	0.001	I
MCP-3 ^b^	−0.50	0.001	I
CXCL1 ^b^	−0.47	0.003	I
MCP-4 ^b^	−0.41	0.011	I
CCL4	−0.33	0.044	I
MCP-1	−0.42	0.008	O
DLCO %			
DCN ^a^	0.37	0.023	R
CPI			
HGF ^b^	0.40	0.013	R
DCN ^a^	−0.38	0.018	R
LAMP3 ^b^	0.40	0.013	I

**Table 6:** Changes in serum proteins from patients with IPF correlates with changes in lung function. Proteins are categorized in two main biological functions, tissue remodeling (R) and inflammation/chemotaxis (I) and overlapping functions (O). Correlations with changes in forced vital capacity (FVC, % predicted), diffusion capacity for carbon monoxide (DLCO, % predicted) and total lung capacity (TLC, % predicted) were calculated using Spearman rank correlation method. Proteins significantly elevated in IPF compared to controls are indicated with ^b^. Proteins observed in ex vivo model are indicated with ^a^.

Change in FVC %	Protein	Rho Coefficient	*p*-Value	Biological Function
	VEGFA ^b^	−0.48	0.003	R
	PDGF subunit B	−0.45	0.005	R
	MMP7 ^a,b^	−0.45	0.006	R
	ANGPT1	−0.38	0.022	R
	NOS3	−0.35	0.033	R
	PD-L1 ^b^	−0.39	0.017	I
	CXCL12	−0.36	0.029	I
	KIR3DL1	−0.35	0.035	I
	CXCL9 ^b^	0.49	0.002	I
	IL12 ^b^	0.45	0.006	I
	PDCD1 ^b^	0.39	0.018	I
	CXCL10 ^b^	0.39	0.019	O
Change in DLCO %				
	NOS3	−0.38	0.021	R
	HGF ^b^	−0.36	0.027	R
	MMP12 ^a,b^	−0.36	0.028	R
	MMP7 ^a,b^	−0.35	0.035	R
	VEGFA ^b^	−0.33	0.045	R
	CD2 7 ^b^	−0.44	0.006	I
	Gal-1 ^b^	−0.38	0.019	I
	IL18 ^b^	−0.35	0.035	I
	CCL4	−0.35	0.035	I
	TNFRSF21 ^b^	−0.33	0.043	I
	KIR3DL1	−0.33	0.045	I
	TNFRSF12A ^b^	−0.69	<0.0001	O
Change in TLC %				
	LAP TGF-beta-1	−0.36	0.029	R
	IL12 ^b^	0.41	0.012	I
	CCL20	0.38	0.021	I
	NCR1	0.35	0.036	I
	CXCL9 ^b^	0.35	0.034	I

**Table 7:** Impact of treatment with antifibrotics on protein concentrations in serum from IPF patients. Patients were divided in three groups according to their treatment status at baseline and at follow-up. *p*-values calculated using Wilcoxon signed-rank test.

Protein	Mean Change (±SD)	Minimum	Maximum	*p*-Value
Untreated baseline and untreated follow-up				
TRAIL	−0.16 ± 0.19	−0.61	0.08	0.014
Untreated baseline and treated follow-up				
EGF	−0.92 ± 0.85	−2.21	0.85	0.003
TNFSF14	−0.75 ± 0.86	−1.87	1.47	0.011
CD40-L	−0.67 ± 0.72	−1.88	0.59	0.011
CASP-8	−0.64 ± 0.56	−1.58	0.16	0.003
FGF2	−0.56 ± 0.45	−1.22	0.3	0.002
CCL3	−0.4 ± 0.38	−1.02	0.13	0.001
IL6	−0.35 ± 0.5	−1.47	0.25	0.033
MUC−16	−0.29 ± 0.38	−1.2	0.3	0.013
CD244	−0.27 ± 0.2	−0.58	0.14	0.001
GZMA	−0.27 ± 0.28	−0.77	0.14	0.003
CD40	−0.26 ± 0.12	−0.46	−0.03	0.0002
LAMP3	−0.26 ± 0.37	−0.86	0.32	0.04
MCP-2	−0.25 ± 0.28	−0.95	0.07	0.006
KLRD1	−0.25 ± 0.24	−0.59	0.19	0.003
VEGFR-2	−0.24 ± 0.11	−0.41	−0.08	0.0002
IL18	−0.24 ± 0.29	−0.71	0.22	0.013
CCL4	−0.23 ± 0.24	−0.67	0.27	0.006
ADA	−0.22 ± 0.22	−0.53	0.3	0.011
TWEAK	−0.21 ± 0.15	−0.44	0.01	0.0005
CD28	−0.21 ± 0.27	−0.69	0.25	0.017
MIC-A/B	−0.21 ± 0.23	−0.59	0.15	0.005
CCL23	−0.19 ± 0.28	−0.66	0.34	0.04
TIE2	−0.18 ± 0.12	−0.42	0	0.0005
FASLG	−0.17 ± 0.13	−0.45	0	0.0005
TRAIL	−0.14 ± 0.21	−0.62	0.14	0.022
KIR3DL1	−0.14 ± 0.18	−0.45	0.15	0.017
ICOSLG	−0.1 ± 0.15	−0.3	0.17	0.04
PD-L2	−0.1 ± 0.17	−0.34	0.32	0.048
TNFRSF21	−0.08 ± 0.13	−0.33	0.17	0.027
IL10	0.21 ± 0.32	−0.39	0.75	0.033
Treated baseline and treated follow-up				
FGF2	−0.31 ± 0.28	−0.83	0.08	0.003
CD28	−0.09 ± 0.15	−0.32	0.11	0.043
CD244	−0.08 ± 0.12	−0.23	0.12	0.027
ICOSLG	−0.06 ± 0.09	−0.2	0.14	0.043
CD8A	0.18 ± 0.19	−0.11	0.58	0.009
MMP12	0.27 ± 0.45	−0.68	1.05	0.043
GZMB	0.33 ± 0.47	−0.12	1.37	0.021
PTN	0.53 ± 0.53	−0.42	1.28	0.007

## Data Availability

The data presented in this study are partly available on reasonable request from the corresponding author. The data are not publicly available due to privacy or ethical restrictions.

## Supplementary Materials

The following are available online at https://www.mdpi.com/article/10.3390/ijms222413421/s1.
