# Supplementary material for: Distal Lung Microenvironment Triggers Release of Mediators Recognized as Potential Systemic Biomarkers for Idiopathic Pulmonary Fibrosis
Source: Int J Mol Sci. 2021 Dec 14;22(24):13421. doi: 10.3390/ijms222413421 (PMC8704101; doi:10.3390/ijms222413421)
Supplement: Supplementary file 1 [file ijms-22-13421-s001.zip › ijms-1492996-supplementary.pdf]

Supplementary material

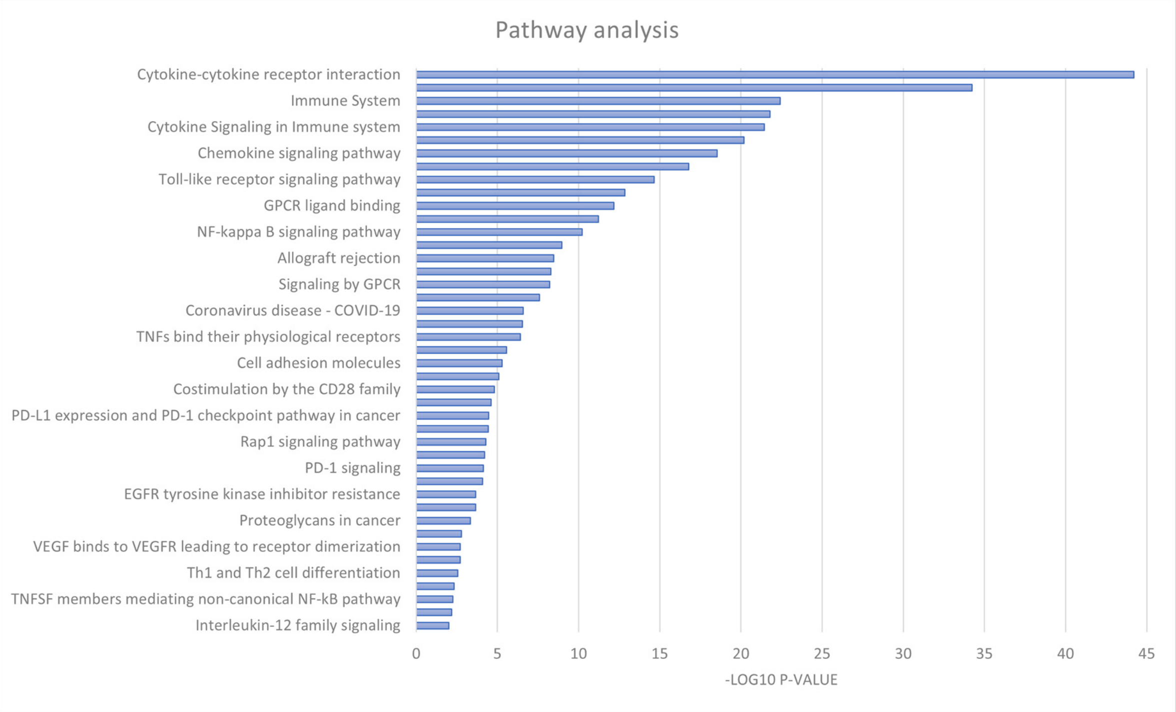

Figure S1. Bioinformatic analysis of elevated proteins in IPF serum at baseline. Pathway analysis depicts an IPF serum profile of associated signaling pathways related to significance (-log10 p-value). Over-representation analysis with Bonferroni correction for multiple testing (significance threshold = 0.01).

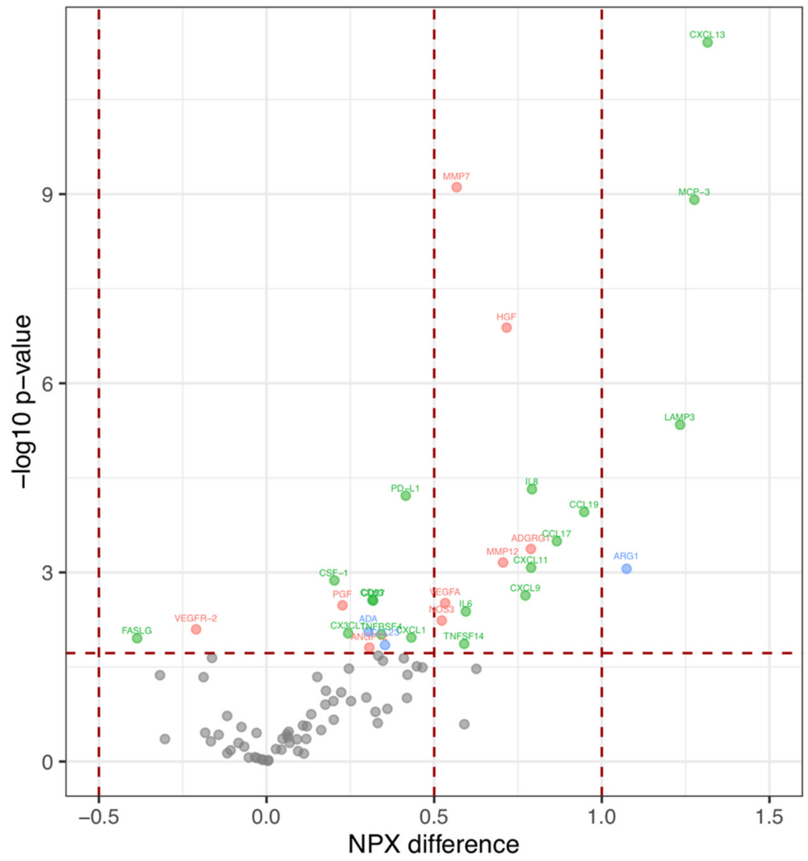

Figure S2. Levels of circulating proteins in IPF patients at follow up versus controls. Volcano plot of the difference in relative protein amount (NPX) (x-axis) by log10 of p-value. **Red:** proteins associated with tissue remodeling; **Green:** proteins associated with inflammation/chemotaxis; **Blue:** proteins with overlapping functions. One-way ANOVA adjusted for age with Benjamini-Hochberg corrected p-value to control a false discovery rate at 5%.

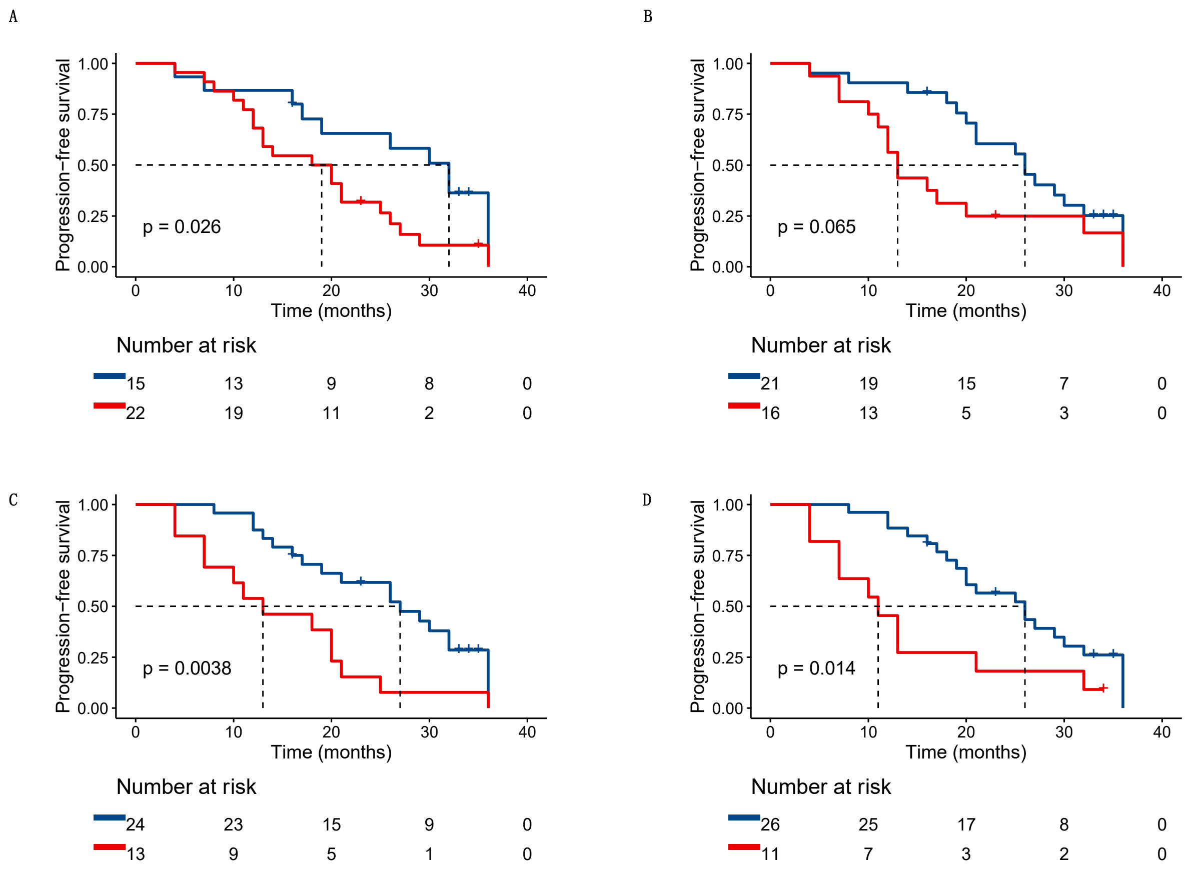

Figure S3. Kaplan-Meier curves for progression-free survival (defined as ≥ 10% relative decline in FVC% or ≥15% relative decline in DLCO%). Lung function tests over a 36 months period from baseline are considered. Groups are stratified by protein change (elevation/reduction) observed at follow up. For each biomarker, **red** indicates the group of patients with elevated levels of respective protein observed at follow up. **Blue** indicates the group of patients with decreasing levels of protein observed at follow up. A) **NOS3,** B) **VEGFA,** C) **CASP-8,** D) **TWEAK**

Table S1. Altered protein levels in IPF patients versus controls at baseline, unadjusted for age. [a] indicate proteins with differentiated expression observed in the ex-vivo model. One-way ANOVA with Benjamini-Hochberg corrected p-value to control a false discovery rate at 5%.

Protein	NPX difference	p-value	FDR adjusted p-value
ADGRG1	1.29	9.52E-16	2.87E-03
PTN	1.25	9.87E-08	1.32E-02
CCL19 [a]	1.22	4.00E-16	2.30E-03
CXCL13 [a]	1.20	1.36E-25	5.75E-04
MCP-3	1.09	1.34E-16	1.72E-03
CXCL9	1.07	9.68E-12	5.75E-03
IL8	1.03	1.43E-13	4.60E-03
LAMP3	0.99	5.19E-09	9.20E-03
MMP12 [a]	0.92	4.64E-12	5.17E-03
ARG1	0.81	5.96E-05	2.36E-02
IL12	0.80	2.04E-08	1.09E-02
CXCL10	0.80	1.82E-07	1.44E-02
CXCL11	0.73	1.47E-07	1.38E-02
CCL17	0.67	2.88E-06	2.07E-02
MMP7 [a]	0.66	2.03E-24	1.15E-03
IL6	0.63	7.19E-07	1.72E-02
HGF	0.61	6.42E-14	4.02E-03
PDCD1	0.56	1.81E-10	6.90E-03
TNFSF14	0.51	4.48E-04	2.76E-02
MCP-4	0.49	1.43E-05	2.18E-02
CRTAM	0.48	2.38E-06	2.01E-02
CCL23	0.47	5.19E-08	1.15E-02
CD27	0.47	2.09E-11	6.32E-03
TNFRSF9 [a]	0.46	6.63E-09	9.77E-03
TNFRSF4	0.44	7.64E-08	1.26E-02
GZMH	0.43	4.09E-04	2.70E-02
TNFRSF12A	0.43	3.92E-10	7.47E-03
TNF	0.43	9.76E-10	8.05E-03
VEGFA	0.43	5.47E-05	2.30E-02
IL10	0.41	3.58E-03	3.22E-02
LAG3	0.41	1.51E-05	2.24E-02
IL12RB1	0.39	7.01E-07	1.67E-02
PGF [a]	0.38	2.47E-14	3.45E-03
CAIX	0.37	3.15E-04	2.64E-02
ANGPT2	0.36	2.09E-06	1.95E-02
PD-L1	0.35	1.29E-08	1.03E-02
ADA	0.35	1.95E-06	1.90E-02

MUC-16	0.35	2.75E-03	3.05E-02
FGF2	0.34	8.83E-04	2.82E-02
CXCL1	0.33	3.00E-04	2.59E-02
KLRD1	0.32	3.47E-03	3.10E-02
CD83	0.32	3.12E-07	1.49E-02
CD28	0.31	6.42E-08	1.21E-02
CD8A	0.31	1.16E-02	3.62E-02
CD40 [a]	0.30	5.70E-07	1.55E-02
Gal-9 [a]	0.30	1.49E-06	1.84E-02
CX3CL1	0.29	5.83E-07	1.61E-02
IL18	0.28	1.25E-02	3.68E-02
CD4 [a]	0.27	2.88E-09	8.62E-03
CCL3	0.26	1.06E-02	3.56E-02
MCP-1	0.25	6.56E-03	3.39E-02
CD70	0.24	3.53E-03	3.16E-02
GZMA [a]	0.24	1.49E-03	2.99E-02
NOS3	0.24	9.24E-03	3.51E-02
Gal-1	0.21	1.21E-06	1.78E-02
CD5	0.19	4.47E-03	3.33E-02
DCN [a]	0.16	1.33E-05	2.13E-02
PD-L2	0.15	1.49E-03	2.93E-02
CSF-1	0.15	8.92E-05	2.47E-02
TNFRSF21 [a]	0.11	7.72E-03	3.45E-02
PDGF subunit B	-0.07	6.18E-05	2.41E-02
VEGFR-2	-0.14	3.76E-03	3.28E-02
ANGPT1	-0.20	1.11E-04	2.53E-02
FASLG	-0.29	1.13E-03	2.87E-02

Table S2. Proteins with differences in concentration in follow up sample from IPF patients versus controls, categorized into two main biological function, tissue remodeling and inflammation/chemotaxis. Three proteins were judged as having overlapping functions. [a] indicate proteins with differentiated expression observed in the ex-vivo model. One-way ANOVA adjusted for age with Benjamini-Hochberg corrected p-value to control a false discovery rate at 5%.

Protein	NPX difference	p-value	FDR adjusted p-value
**Tissue remodeling**			
ADGRG1	0.79	4.24E-04	5.75E-03
HGF	0.72	1.31E-07	2.30E-03
MMP12[a]	0.71	6.96E-04	6.32E-03
MMP7[a]	0.57	7.76E-10	1.15E-03
VEGFA	0.53	3.07E-03	1.03E-02
NOS3	0.52	5.80E-03	1.21E-02
ANGPT2	0.31	1.56E-02	1.72E-02
PGF[a]	0.23	3.33E-03	1.09E-02
VEGFR-2	-0.21	8.01E-03	1.26E-02
**Inflammation/Chemotaxis**			
CXCL13[a]	1.32	3.93E-12	5.75E-04
MCP-3	1.28	1.23E-09	1.72E-03
LAMP3	1.23	4.55E-06	2.87E-03
CCL19[a]	0.95	1.10E-04	4.60E-03
CCL17	0.87	3.20E-04	5.17E-03
IL8	0.79	4.81E-05	3.45E-03
CXCL11	0.79	8.40E-04	6.90E-03
CXCL9	0.77	2.32E-03	8.62E-03
IL6	0.59	4.17E-03	1.15E-02
TNFSF14	0.59	1.36E-02	1.61E-02
CXCL1	0.43	1.08E-02	1.49E-02
PD-L1	0.42	6.07E-05	4.02E-03
TNFRSF4	0.34	9.66E-03	1.44E-02
CD27	0.32	2.80E-03	9.77E-03
CD83	0.32	2.75E-03	9.20E-03
CX3CL1	0.24	9.28E-03	1.38E-02

CSF-1	0.20	1.34E-03	8.05E-03
FASLG	-0.39	1.11E-02	1.55E-02
**Overlapping functions**			
ARG1	1.07	8.77E-04	7.47E-03
CCL23	0.35	1.42E-02	1.67E-02
ADA	0.30	8.74E-03	1.32E-02

Table S3. Altered protein levels in IPF patients versus controls at follow up, unadjusted for age. [a] indicate proteins with differentiated expression observed in the ex-vivo model. One-way ANOVA with Benjamini-Hochberg corrected p-value to control a false discovery rate at 5%.

Protein	NPX difference	p-value	FDR adjusted p-value
PTN	1.53	2.21E-10	6.90E-03
CXCL13[a]	1.43	4.85E-26	5.75E-04
CXCL9	1.25	8.43E-13	3.45E-03
ADGRG1	1.17	3.51E-14	2.30E-03
CCL19 [a]	1.14	2.20E-12	4.02E-03
MCP-3	1.04	2.15E-14	1.72E-03
MMP12 [a]	0.98	2.85E-12	4.60E-03
IL8	0.86	1.17E-11	6.32E-03
CXCL11	0.85	1.93E-08	7.47E-03
LAMP3	0.84	4.92E-07	1.09E-02
CXCL10	0.79	1.42E-06	1.38E-02
IL6	0.70	1.23E-07	9.77E-03
CCL17	0.69	3.90E-06	1.55E-02
IL12	0.68	5.91E-06	1.61E-02
MMP7 [a]	0.65	3.87E-23	1.15E-03
HGF	0.64	2.76E-13	2.87E-03
ARG1	0.63	1.45E-03	2.76E-02
GZMH	0.62	6.74E-06	1.67E-02
IL10	0.60	9.40E-04	2.70E-02
PDCD1	0.52	3.29E-08	8.05E-03
CD27	0.49	9.09E-12	5.75E-03
CRTAM	0.48	7.21E-06	1.72E-02
MCP-4	0.44	9.79E-05	2.18E-02
LAG3	0.43	9.83E-06	1.84E-02
NOS3	0.43	2.30E-04	2.36E-02
TNFRSF4	0.42	7.52E-07	1.32E-02
TNFRSF9 [a]	0.41	5.42E-07	1.15E-02
VEGFA	0.41	2.26E-04	2.30E-02

TNFRSF12A	0.41	6.40E-08	8.62E-03
CCL23	0.39	1.83E-05	1.90E-02
TNF	0.37	7.20E-07	1.26E-02
PGF [a]	0.36	7.64E-12	5.17E-03
ANGPT2	0.35	9.57E-06	1.78E-02
PD-L1	0.33	3.59E-07	1.03E-02
MUC-16	0.32	1.09E-02	3.10E-02
CD8A	0.32	1.08E-02	3.05E-02
Gal-9 [a]	0.31	1.59E-06	1.44E-02
CX3CL1	0.29	6.91E-07	1.21E-02
CXCL1	0.29	5.40E-03	2.87E-02
KLRD1	0.28	1.72E-02	3.33E-02
IL12RB1	0.28	2.60E-04	2.47E-02
CAIX	0.27	6.39E-03	2.93E-02
IL7	0.24	1.45E-02	3.22E-02
CD83	0.23	3.03E-04	2.53E-02
ADA	0.23	1.59E-03	2.82E-02
CD40[a]	0.21	5.17E-04	2.59E-02
MCP-1	0.21	2.45E-02	3.51E-02
CD4[a]	0.20	2.27E-05	1.95E-02
GZMA[a]	0.19	1.40E-02	3.16E-02
CD70	0.19	3.13E-02	3.68E-02
CD28	0.16	8.79E-03	2.99E-02
DCN[a]	0.15	5.65E-05	2.01E-02
CSF-1	0.15	8.93E-05	2.13E-02
Gal-1	0.14	7.66E-04	2.64E-02
PD-L2	0.11	1.73E-02	3.39E-02
PDGF subunit B	-0.07	2.34E-04	2.41E-02
TIE2	-0.10	1.90E-02	3.45E-02
TWEAK	-0.13	3.26E-02	3.74E-02
CD244	-0.14	1.51E-02	3.28E-02
TRAIL	-0.23	1.09E-04	2.24E-02
VEGFR-2	-0.27	8.69E-08	9.20E-03
ANGPT1	-0.27	2.82E-06	1.49E-02
EGF	-0.31	3.05E-02	3.62E-02
CD40-L	-0.34	2.48E-02	3.56E-02
FASLG	-0.37	7.15E-05	2.07E-02

Table S4. Correlations between protein levels in the follow up serum sample from IPF patients and disease severity, defined by forced vital capacity (FVC, % predicted), total lung capacity (TLC, % predicted), diffusion capacity for carbon monoxide (DLCO, % predicted) and composite physiological index (CPI). Proteins are categorized in two main biological functions, tissue remodeling (R) and inflammation/chemotaxis (I). Three proteins have overlapping functions (O). P and r values were determined using Spearman rank correlation method.

FVC%	Rho coefficient	p-value	Biological function
MMP7	-0.52	0.001	R
PDGF subunit B	-0.50	0.002	R
PTN	0.43	0.008	R
MCP-3	-0.49	0.002	I
LAMP3	-0.45	0.006	I
IL-8	-0.40	0.015	I
CSF-1	-0.38	0.020	I
MCP-1	-0.38	0.021	I
KIR3DL1	-0.37	0.024	I
TRAIL	-0.45	0.006	O
TWEAK	-0.37	0.023	O
ARG1	-0.35	0.033	O

**TLC%**			
MMP7	-0.39	0.017	R
PDGF subunit B	-0.33	0.048	R
HGF	-0.35	0.036	R
PTN	0.39	0.016	R
MCP-3	-0.46	0.004	I
IL8	-0.40	0.015	I
TNFSF14	-0.40	0.014	I
CCL3	-0.34	0.037	I
KIR3DL1	-0.34	0.042	I
ARG1	-0.40	0.015	O
GZMB	-0.37	0.026	O
**DLCO%**			
FGF2	-0.37	0.026	R
PDGF subunit B	-0.36	0.027	R
MUC-16	-0.36	0.03	I
IL6	-0.33	0.047	I
ARG1	-0.39	0.018	O
TRAIL	-0.36	0.028	O
CASP-8	-0.36	0.030	O
TWEAK	-0.33	0.044	O
**CPI**			
MMP7	0.46	0.004	R
PDGF subunit B	0.45	0.006	R
FGF2	0.33	0.048	R
DCN	-0.34	0.037	R
PTN	-0.37	0.026	R
MCP-3	0.42	0.010	I
LAMP3	0.36	0.028	I
ARG1	0.41	0.011	O
TRAIL	0.39	0.017	O
CASP-8	0.36	0.029	O
TWEAK	0.34	0.038	O

Table S5: Progressive patients demonstrated increases in protein levels as opposed to patients who remained stable. P-values calculated using Wilcoxon signed-rank test.

Protein	Progressive patients (mean difference) (95%CI)	Stable patients (mean difference) (95%CI)	p-value	Biological function
NOS3	0.44 (0.002-0.89)	-0.04 (-0.21-0.12)	0.013	R
HGF	0.14 (-0.04-0.33)	-0.1 (-0.25 -0.05)	0.036	R
VEGFA	0.08 (-0.07-0.23)	-0.12 (-0.24-(-0.0001)	0.033	R
MMP7	0.04 (-0.03-0.12)	-0.06 (-0.13-0.005)	0.031	R
TNFRSF12A	0.15 (0.007-0.30)	-0.19 (-0.35-(-0.04))	0.0008	O
CD8a	0.13 (0.004-0.25)	-0.09 (-0.24-0.06)	0.026	I
CD27	0.07 (-0.002-0.15)	-0.04 (-0.11-0.03)	0.031	I
CXCL12	0.06 (-0.04-0.16)	-0.10 (-0.21-0.008)	0.031	I

KLRD1	0.03 (-0.14-0.19)	-0.13 (-0.29-0.02)	0.044	I
Gal-1	0.00012 (-0.09-0.09)	-0.13 (-0.21-(-0.04))	0.044	I
CASP-8	-0.035(-0.31-0.24)	-0.49 (-0.79-(-0.19))	0.022	O

Table S6. Predictive value of baseline levels and the differences in protein levels observed at follow up for progression over 36 months assessed by Cox proportional hazards model. Covariates adjusted for in the multivariate analysis included age, gender, FVC% and DLCO% at baseline.

**Variables**	**HR**	**Univariate analysis 95% CI**	**p-value**	**HR**	**Multivariate analysis * 95% CI**	**p-value**
Age (years)	0.99	0.96-1.04	0.82			
Gender, female	0.77	0.31-1.90	0.57			
FVC%	1.00	0.98-1.02	0.97			
DLCO%	1.01	0.97-1.04	0.76			

MMP7, baseline	0.36	0.05-2.74	0.33	0.20	0.15-2.51	0.21
TNFRSF12A, baseline	0.28	0.09-0.99	0.047	0.18	0.04-0.77	0.02
TWEAK, baseline	0.30	0.08-1.20	0.08	0.13	0.02-0.74	0.02
HGF, baseline	0.64	0.28-1.49	0.29	0.65	0.27-1.57	0.34
VEGFA, baseline	0.91	0.49-1.69	0.76	0.91	0.49-1.71	0.77
NOS3, baseline	1.23	0.61-2.49	0.57	1.27	0.56-2.88	0.57
GAL9, baseline	0.87	0.26-2.95	0.82	0.76	0.20-2.85	0.68
CD40, baseline	0.49	0.17-1.43	0.19	0.47	0.15-1.51	0.21
CXCL13, baseline	1.48	0.83-2.64	0.19	1.65	0.90-3.02	0.10
**Differences in protein expression – time to progression (36 months)**						
ΔMMP7	35.5	3.01-418	0.005	63.0	4.36-917.7	0.002
ΔTNFRSF12A	2.94	1.09-7.94	0.03	3.33	1.24-8.92	0.02
ΔTWEAK	4.15	0.97-17.7	0.05	7.87	1.71-36.2	0.008
ΔHGF	2.53	0.95-6.73	0.06	2.4	0.90-6.31	0.08
ΔVEGFA	3.60	1.12-11.5	0.03	4.19	1.27-13.8	0.02
ΔNOS3	1.77	1.15-2.71	0.01	1.70	1.12-2.58	0.01
ΔGAL9	3.23	0.64-16.4	0.16	2.81	0.57-13.9	0.20
ΔCD40	5.3	1.13-24.9	0.03	9.24	1.95-43.8	0.005
ΔCXCL13	2.05	1.05-4.00	0.04	1.99	1.03-3.88	0.04
